# A case report of a thymic neuroblastoma associated with syndrome of inappropriate secretion of antidiuretic hormone: Ten-year follow-up results after surgical treatment

**DOI:** 10.1016/j.ijscr.2019.03.056

**Published:** 2019-04-05

**Authors:** Yukitoshi Satoh

**Affiliations:** Department of Thoracic Surgery, Kitasato University School of Medicine, 1-15-1 Kitasato, Minami-ku, Sagamihara-shi, Kanagawa 252-0374, Japan

**Keywords:** SIADH, syndrome of inappropriate secretion of antidiuretic hormone, Thymus, Neuroblastoma, Surgery, Survival

## Abstract

**Introduction:**

Thymic neuroblastoma with syndrome of inappropriate secretion of antidiuretic hormone (SIADH) in adults is an extremely rare and malignant neoplasm. Although the tumor is known to grow rapidly and have a poor prognosis, its etiology is still uncertain. In 2009, we described a case of thymic neuroblastoma with SIADH. We report here the clinical course over the last 10 years.

**Presentation of case:**

A 70-year-old Japanese male with a history of thymic neuroblastoma with SIADH was regularly followed up on an out-patient basis after complete surgical resection for more than 10 years. He remains free of the disease and asymptomatic.

**Discussion and conclusion:**

Complete surgical removal is considered as one of the treatment options for this tumor if clearly separated from the surrounding tissue with no invasion.

## Highlights

• A rare case of thymic neuroblastoma in an adult associated with the syndrome of inappropriate secretion of antidiuretic hormone (SIADH).
• Histologically and hormonally confirmed for a thymic neuroblastoma in an adult associated with SIADH.
• Long-term follow-up CT scans reveal his free of disease.
• The thymic neuroblastoma is most effectively treated with surgical resection and follow-up examinations.
• Complete surgical removal is considered to be one of options for treatment of this tumor that is clearly separated from the surrounding tissue with no invasion.

## 1 Introduction

In 2009, we reported an extremely rare case of thymic neuroblastoma in an adult with syndrome of inappropriate secretion of antidiuretic hormone (SIADH) [1]. Till date, few cases of thymic neuroblastoma with SIADH have been reported in the English literature [2,3]. Although neuroblastomas are known to be malignant, grow rapidly, and have a poor prognosis, the etiology of thymic neuroblastoma is still uncertain. Moreover, limited long-term prognostic data on the disease in adults are available, and standard therapy is yet to be established [1]. We describe here the clinical course of neuroblastoma with SIADH over a period of 10 years; the patient achieved a successful outcome and had no recurrence after surgery. We focus on the management and surgical assessment. This work has been reported in line with the SCARE criteria [4].

## 2 Presentation of case

**Fig. 1:**
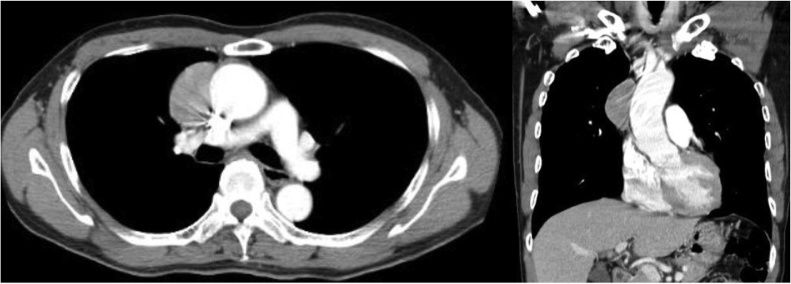
Contrast computed tomography scan of the chest: 47-mm solid mass with a clear rim in the anterior mediastinum.

In 2008, a 60-year-old Japanese male patient was admitted to our hospital for further examination and treatment of an abnormal shadow seen on his chest x-ray during a regular health check-up. An enhanced chest computed tomography scan revealed a 47-mm solid mass with a clear rim in the anterior mediastinum (Fig. 1). Magnetic resonance imaging using intravenous contrast showed iso-intensity and high intensity of the mass on T1- and T2-weighted images, respectively. Laboratory findings showed a serum sodium concentration of 119 mEq/L, plasma osmolality of 261 mEq/L, and an elevated plasma antidiuretic hormone level of 6.4/L. A diagnosis of thymoma with SIADH was suspected, and the patient underwent total thymectomy. Based on the microscopic findings with immunohistochemistry, the final diagnosis was thymic neuroblastoma. The histologic pattern confirmed it as a poorly differentiating neuroblastoma based on the classification according to the new World Health Organization system [5]. The tumor had a thin, fibrous capsule and showed no invasion into the surrounding atrophic thymic tissue. His serum sodium levels and blood osmolality quickly returned to normal after surgical excision of the tumor. The patient was provided detailed information about the disease. We recommended adjuvant therapy since the disease is considered malignant with a poor prognosis; however, the patient did not wish to undergo therapy after surgery. Therefore, he was regularly followed up on an outpatient basis with periodic computed tomography scans and laboratory studies including blood analysis and urinalysis once a year. At the 10-year follow-up after surgery, the 70-year-old patient remains free of the disease and is asymptomatic. Witten informed consent was obtained from this patient for the publication of this report.

## 3 Discussion

Neuroblastoma is considered a pediatric malignancy since over 95% of the cases are diagnosed in children aged less than 10 years [6]. These tumors have a very broad spectrum of clinical manifestations, which can include spontaneous regression, maturation to a benign ganglioneuroma, or aggressive disease with metastatic dissemination leading to death [3]. However, due to its extreme rarity in adults, no standard therapy has been established despite its poor prognosis [7]. Moreover, unlike the present case, there are no reported cases of long-term survival for more than 10 years without recurrence or metastasis [8].

In adult neuroblastomas, localized tumors can frequently recur, and reports suggest that recurrence or metastasis inevitably appears following treatment with surgery alone or combination of either radiation or chemotherapy [6]. However, the natural course of neuroblastoma in the elderly differs from that in children. In infant populations, the tumor has a more indolent behavior, but it has a poor prognosis in the elderly [3]. Since primary neuroendocrine neoplasms occurring in the mediastinum, particularly in the thymus, are very rare, factors related to long-term prognosis and standard therapy are yet to be established [1,8]. In the present case of a tumor originating from the thymus, the patient did not wish to undergo postoperative adjuvant therapy. However, the tumor was completely excised and was found to have a fibrous capsule with no invasion into the surrounding tissues on histology. We regularly followed up our patient for about 10 years after surgery.

## 4 Conclusion

In the present case, complete surgical removal is considered to be an option for the treatment of this tumor when it is clearly separated from the surrounding tissues and with no invasion. However, further investigations are necessary to confirm whether it could indeed be a treatment strategy in this disease.

## Please state any conflict of interest

The author declares no conflicts of interests.

## Please state any sources of funding for your research

The author did not receive any fund.

## Ethical approval

This case report is exempted from ethical approval by our institution.

## Consent

The author states that they have written and signed consent from the patient to publish this report.

## Author contribution

Yukitoshi Satoh: Conceptualization, Methodology, Validation, Investigation, Writing – original draft, Writing – review and editing, Visualization, Supervision.

## Registration of research studies

The author does not need to register this work.

## Guarantor

Dr. Yukitoshi Satoh.

## Provenance and peer review

Not commissioned, externally peer-reviewed.
